# Growth hormone in the presence of laminin modulates interaction of human thymic epithelial cells and thymocytes in vitro

**DOI:** 10.1186/s40659-016-0097-0

**Published:** 2016-09-02

**Authors:** Marvin Paulo Lins, Larissa Fernanda de Araújo Vieira, Alfredo Aurélio Marinho Rosa, Salete Smaniotto

**Affiliations:** Laboratory of Cell Biology, Institute of Biological Sciences and Health, Federal University of Alagoas, Maceió, Alagoas Brazil

**Keywords:** Thymocyte, Growth hormone, Laminin, Thymic epithelial cells

## Abstract

**Background:**

Several evidences indicate that hormones and neuropeptides function as immunomodulators. Among these, growth hormone (GH) is known to act on the thymic microenvironment, supporting its role in thymocyte differentiation. The aim of this study was to evaluate the effect of GH on human thymocytes and thymic epithelial cells (TEC) in the presence of laminin.

**Results:**

GH increased thymocyte adhesion on BSA-coated and further on laminin-coated surfaces. The number of migrating cells in laminin-coated membrane was higher in GH-treated thymocyte group. In both results, VLA-6 expression on thymocytes was constant. Also, treatment with GH enhanced laminin production by TEC after 24 h in culture. However, VLA-6 integrin expression on TEC remained unchanged. Finally, TEC/thymocyte co-culture model demonstrated that GH elevated absolute number of double-negative (CD4^−^CD8^−^) and single-positive CD4^+^ and CD8^+^ thymocytes. A decrease in cell number was noted in double-positive (CD4^+^CD8^+^) thymocytes.

**Conclusions:**

The results of this study demonstrate that GH is capable of enhancing the migratory capacity of human thymocytes in the presence of laminin and promotes modulation of thymocyte subsets after co-culture with TEC.

## Background

Leukocytes differentiate from multipotent cells in the bone marrow known as hematopoietic stem cells (HSCs). T cells fully maturate in thymus gland, a primary lymphoid organ that is involved in the differentiation of these cells [1]. The organization of thymic stroma provides specialized niches that ensure appropriate development of T cell progenitors [2]. This microenvironment comprises distinct cell types, including thymic epithelial cells (TEC), dendritic cells, macrophages and fibroblasts. Moreover, such tridimensional cellular organization is intermingled with an extracellular matrix (ECM)-containing network [3].

Progenitor cells enter the thymus through blood vessels at the cortico-medullary junction and migrate across different thymic regions, from the subcapsular cortex to deep cortex, toward the medulla, where they differentiate [4]. During this migration, thymocytes interact with stromal cells, especially with TEC through cytoplasmic projections and membrane receptors [2]. Thymocyte maturation is based on a series of intracellular signaling events that regulate differentiation, proliferation and cell survival [3]. This process may be monitored through the presence or absence of cell surface markers such as CD4 and CD8 glycoproteins. The most immature thymocytes do not express CD4 or CD8 and are called double-negative cells (DN). Following their development, DN cells express both CD4 and CD8 to become double-positive cells (DP). Finally, upon maturation the cells differentiate into single-positive (SP) thymocytes expressing either CD4 or CD8 when they emigrate from the thymus and populate the peripheral lymphoid organs [5–7].

It is well established that immune cells secrete and/or possess receptors for various hormones, indicating bidirectional communication between the neuroendocrine and immune systems [8]. Growth hormone (GH) is one of the first known pituitary peptides that have profound effects on regulation of the immune system in vivo through endocrine, paracrine and autocrine mechanisms, along with some cytokines [9].

Numerous thymic functions are modulated by GH. For example, GH acts on thymocytes, increasing proliferation of these cells and stimulates TEC growth, which are essential for T cell differentiation. Furthermore, GH increases the production of extracellular matrix molecules by TEC and secretion of soluble peptides, such as cytokines and chemokines, directly involved in intrathymic migration of thymocytes and their export to the periphery [10–12].

In the last years, effects of GH on thymic cells have been studied, however many points need to be clarified. In this study, we investigated the in vitro role of GH on adhesive and migratory capacities of human thymocytes. Furthermore, laminin production and VLA-6 integrin expression were assessed on TEC after GH-treatment. Finally, thymocyte subsets were examined after contact with TEC (co-culture) in the presence of GH.

## Results

### GH enhances thymocyte adhesion on substrates

In the context of thymocyte differentiation inside thymus gland, these cells interact directly with ECM, receiving signals to maturation. Thus, thymocytes adhere to these molecules, utilizing membrane receptors, and this adhesion is induced or impaired for various hormones [13].

**Fig. 1:**
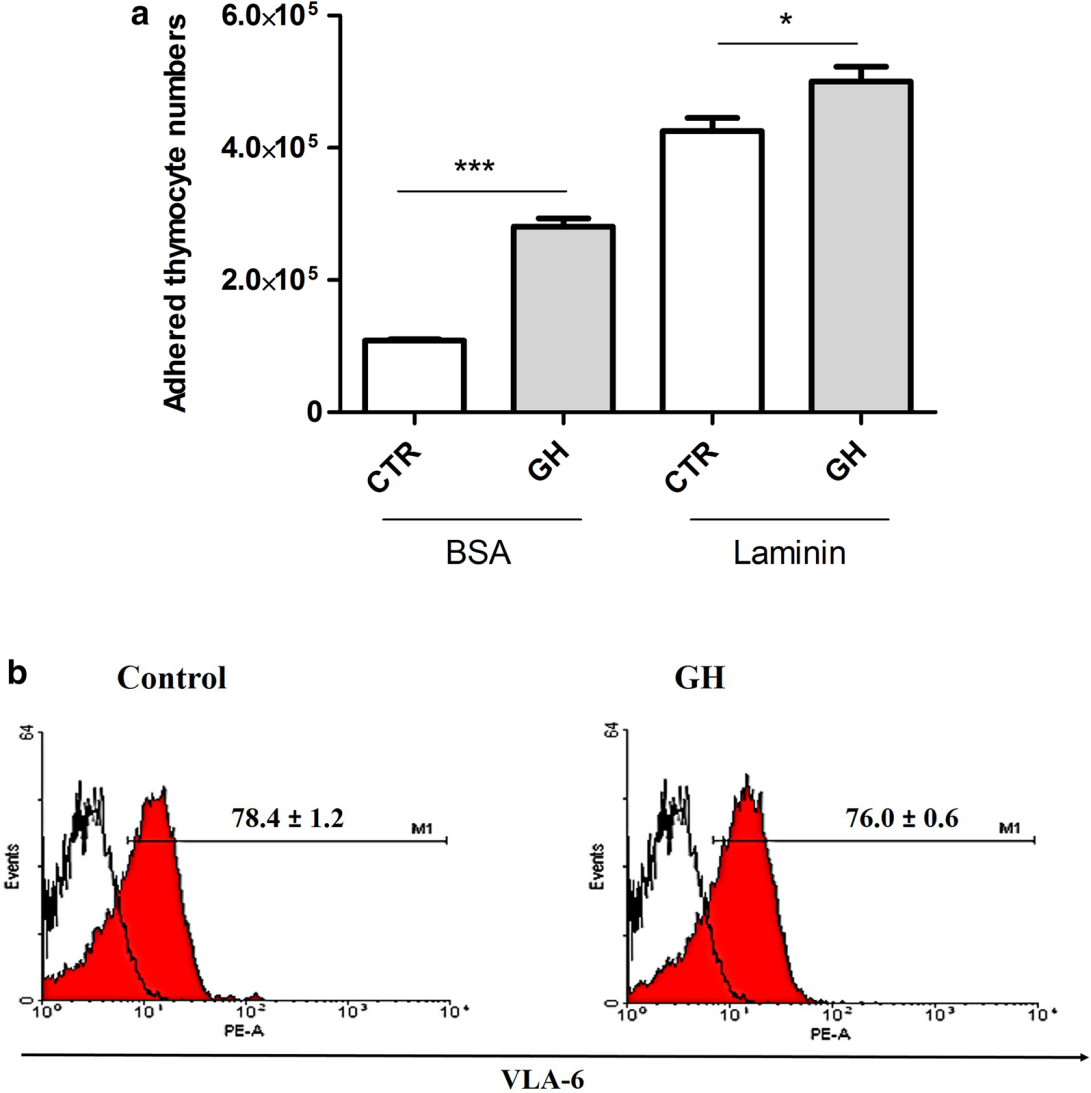
GH promotes thymocyte adhesion to laminin. Thymocytes were exposed to GH (100 ng/mL) for 1 h and were then allowed to adhere on BSA or laminin substrata. **a** Absolute number of adhered cells, revealing that GH increases the number of adhered thymocytes on BSA and laminin substrata (n = 6/group). **b** Representative histograms demonstrate VLA-6 expression on thymocytes after 1 h of adhesion (n = 6/group). *Filled curves* represent cells positive for VLA and *white curve* represents the Ig isotype control. Values are expressed as mean ± SEM. *p ≤ 0.05 and ***p < 0.001

To examine whether thymocyte adhesion on ECM-composed substrata was modified by GH treatment, these cells were allowed to adhere for 1 h in Petri dishes coated with laminin or BSA. It was observed that on BSA substrate, GH increases number of adhered thymocytes. On laminin substrate, number of adhered cells was greater than that on BSA substrate, and GH was able to further stimulate thymocyte adhesion on laminin (Fig. 1a). When investigating expression of VLA-6, a laminin receptor, on adhering cells, it was found that adhered thymocytes in the presence of GH had same expression levels of this integrin as adhered cells in the absence of GH (Fig. 1b).

### Thymocyte migration through laminin is improved by GH

**Fig. 2:**
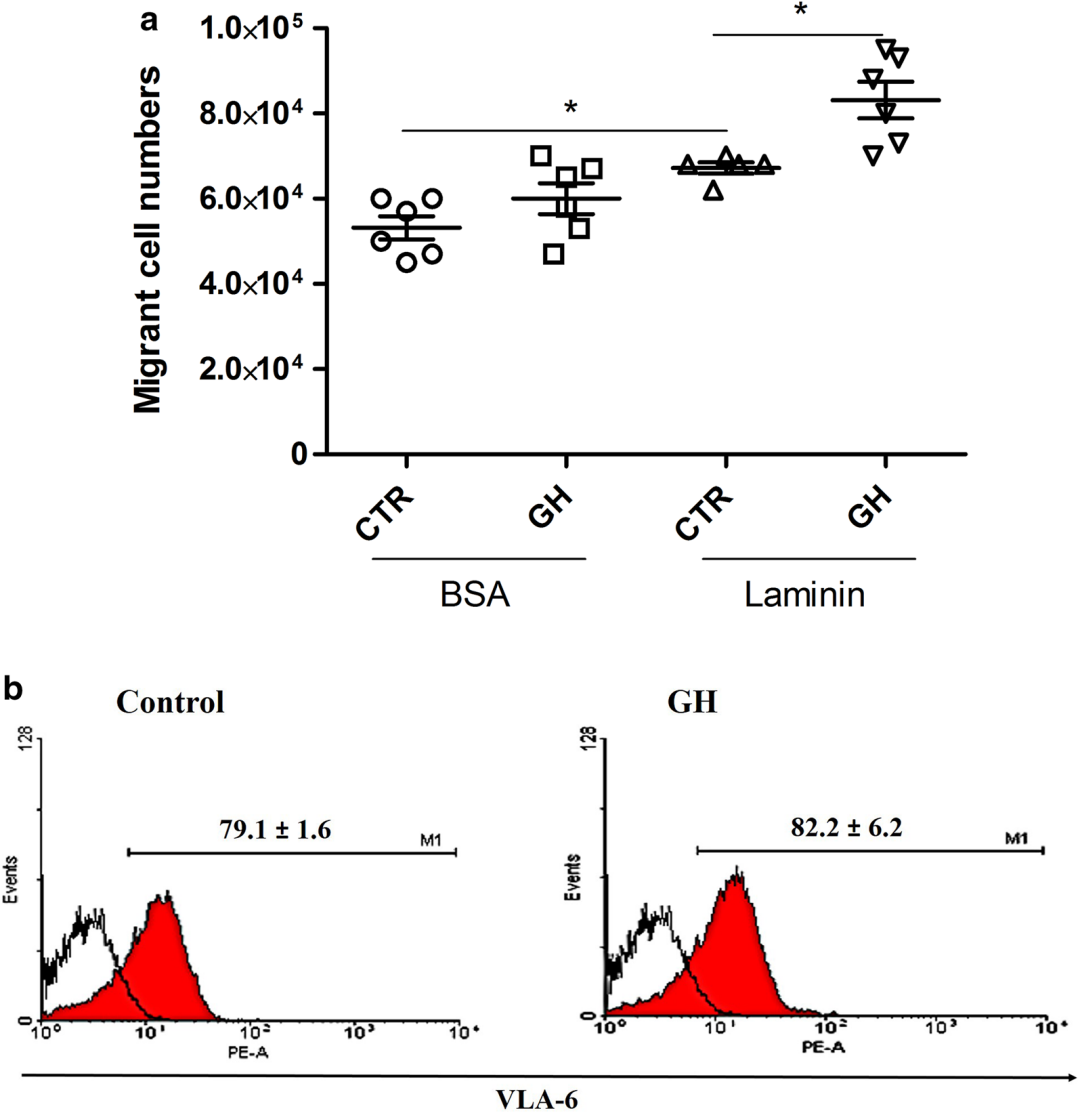
GH improves thymocyte migration through laminin-coating. After 3 h of migration in BSA or laminin-coated transwell. **a** Absolute number of migrant cells, indicating that GH increases thymocyte migration on laminin substrate. **b** Representative histograms demonstrate VLA-6 expression on thymocytes after migration. *Filled curves* represent VLA positive cells and *white curve* represents Ig the isotype control. Values are expressed as mean ± SEM, n = 6 *p ≤ 0.05

Cell migration is a multistep process involving changes in the cytoskeleton, cell-substrate adhesions and ECM [14]. Once that GH promotes thymocyte adhesion, mainly on laminin, it was evaluated whether GH modulates thymocyte migration on transwell inserts. After cell migration for 3 h, it was found that GH maintains thymocyte migration at normal rates. However, on laminin coating, the number of migrating cells in GH-treated group was higher than the control (Fig. 2a). Nevertheless, it was observed that expression of VLA-6, in both situations, was unchanged (Fig. 2b).

### Increased production of laminin by GH-treated TEC

**Fig. 3:**
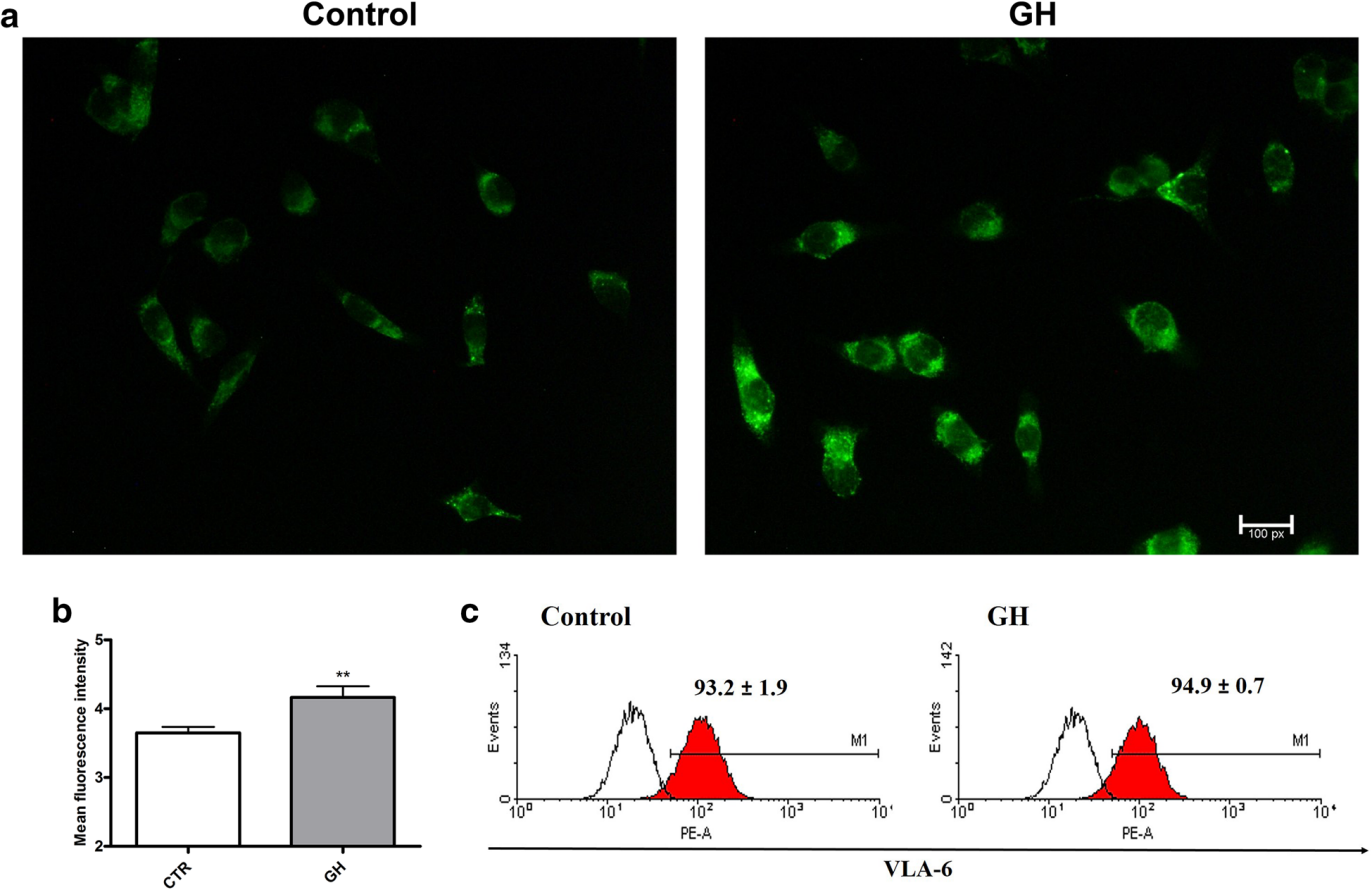
Laminin production by TEC after GH-treatment. TECs were plated in labtek chamber slides, treated with GH (100 ng/mL) for 24 h and then analyzed by fluorescence microscopy. **a** Photomicrographs show the production of laminin ascertained by immunofluorescence and fluorescence microscopy analysis. **b** *Bars* correspond to the quantitative analysis of laminin production in TEC in selected microscopic fields. Results are expressed as pixels/μm^2^. GH-treated cells increase laminin deposition. **c** Cytofluorometric profiles of TEC immunolabeled with anti-CD49f mAb, which defines the alpha chain of integrin VLA-6, the main receptor for laminin. Filled curves represent positive cells for VLA and white curve represents the Ig isotype control. Values correspond to mean ± SEM of three independent experiments, **p ≤ 0.01

Next assessments were focused on human TEC and its laminin production after GH treating, since they are major cell type of the thymus and the main source of ECM molecules [13]. Thus, an immunocytochemistry assay was performed. Qualitative analysis showed that GH treatment increased laminin production (Fig. 3a). This was confirmed, quantitatively, by fluorescence intensity, which demonstrated a significant increase in laminin accumulation (Fig. 3b).

Considering the differences observed in laminin production patterns, the membrane expression of the laminin receptor was evaluated in TEC after exposure to GH. The expression of VLA-6 on TEC was essentially the same in control versus GH-treated groups (Fig. 3c).

### GH promotes modulation in thymocyte subsets after co-culture with TEC

ECM proteins, such as laminin, have been shown to actively contribute to the interaction of developing T cells with the thymic epithelium during the intrathymic migration of thymocytes. Moreover, thymocyte/TEC interaction is also a two-way process in which the functioning of TEC is dependent on the influence of thymocytes [15]. For this propose, human thymocyte subsets after contact with TEC were evaluated in a co-culture model in vitro, and the contribution of GH to the modulation of thymocyte subsets was examined. Fresh thymocytes were added on the TEC monolayer, with or without GH, and analyzed after 24 h to determine the absolute numbers of all thymocyte subsets.

**Fig. 4:**
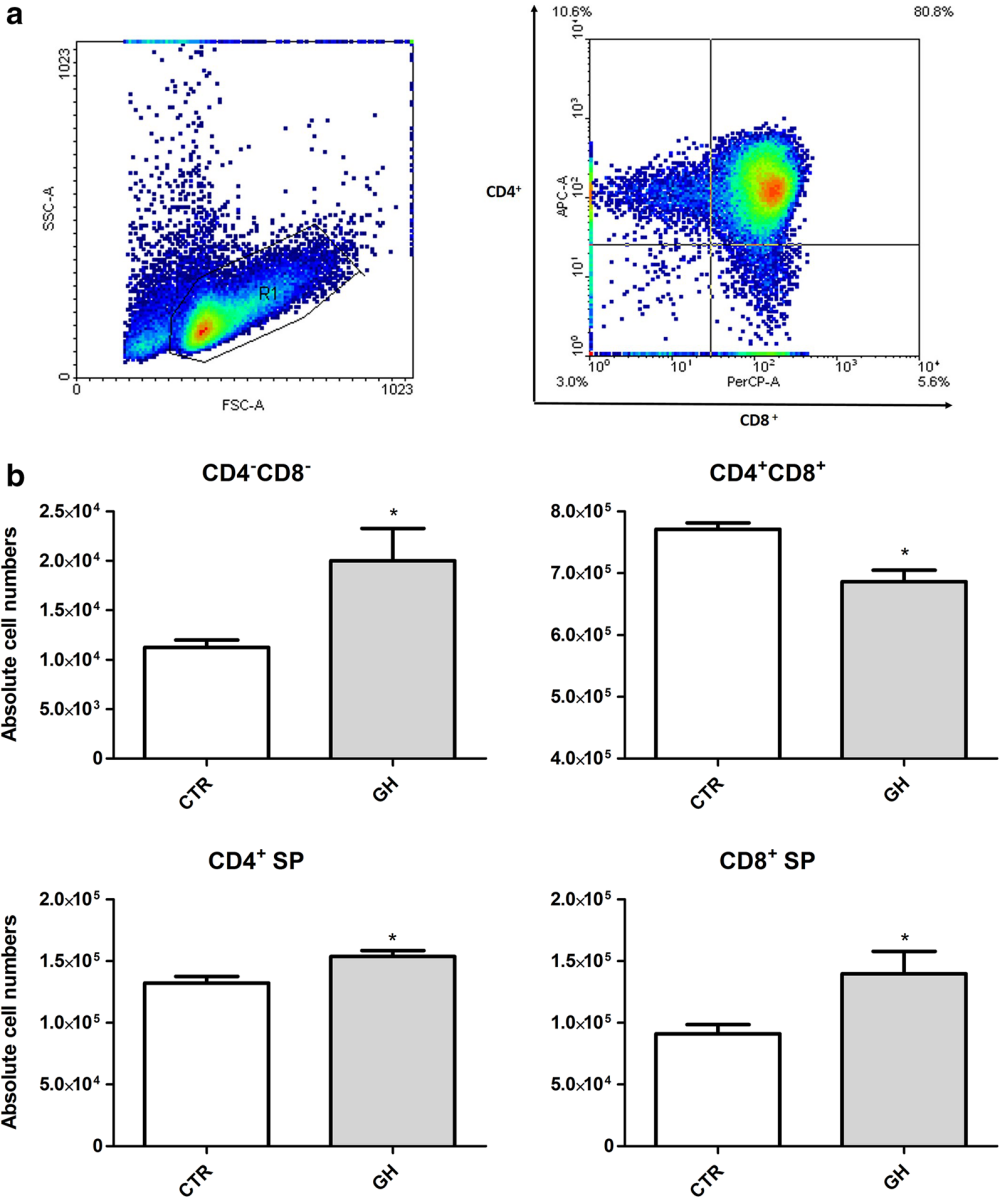
Modulation of thymocyte subsets by GH-treatment and after contact with TEC. At the end of 24 h of contact with TEC in the presence or absence of GH, thymocytes were recovered and stained with CD4/CD8 mAbs, fixed, and analyzed by flow cytometry. In **a** viable thymocytes were selected and quadrants were used to individualize subsets, showing the cell percentage in each. In **b** *bars* show the absolute numbers of CD4/CD8-defined thymocyte subsets. GH induced distinct modulation in subsets, increasing CD4^−^CD8^−^, CD4^+^ and CD8^+^ subsets, and decreasing the CD4^+^CD8^+^ subset. Results are expressed as mean ± SEM (n = 6/group). *p ≤ 0.05 and **p ≤ 0.01

Dotplots were first obtained to demonstrate the total number of thymocytes and the percentage of cells in each thymocyte subset (double-negative, double-positive, CD4^+^ single-positive and CD8^+^ single-positive), as shown in Fig. 4a. Absolute cell numbers were then compared between the control and GH-treated groups. The numbers of double-negative (CD4^−^CD8^−^) thymocytes were increased after contact with TEC in the presence of GH. This effect also was observed in the mature subsets, CD4^+^ single-positive and CD8^+^ single-positive thymocytes. Interestingly, double-positive (CD4^+^CD8^+^) thymocytes showed reduced cell numbers in the GH-treated group (Fig. 4b).

## Discussion

The thymus is an organ for T cell differentiation and selection. Developing thymocytes migrate across the thymus to undergo positive and negative selection in the cortex and medulla, respectively. In these migration process, several events occur which thymocytes adhere to their surrounding ECM and various types of stromal cells within the thymus. These stromal cells produce ECM, including laminin, which mediates cell–cell contact [16]. Laminin is a heterotrimer of alpha, beta and gamma chains. Combinations of five α-, four β- and three γ-chains give rise to at least 16 different laminin isoforms in mammals. Laminin isoforms can drive numerous processes in cells, including cell adhesion, differentiation, proliferation, survival/apoptosis and migration. All these biological processes require the participation of integrins, which are the major receptors for ECM proteins [17]. In addition to these intrinsic factors, it is known that the thymus is a target of neuroendocrine control through the expression of many types of receptors for hormones and neuropeptides, which act in endocrine, paracrine and autocrine manners. In this aspect, GH exerts pleiotropic actions on the thymic microenvironment [18].

In this study, it was demonstrated that some activities of human thymocytes and TEC are modulated by GH. These findings are consistent with previous studies obtained in murine models from other groups and of our group [19–21]. To promote its effects on thymic cells, the molecule binds to GH receptor present on TEC and thymocyte surface [22]. It was found that GH stimulates human thymocytes to adhere on BSA substrate and also on laminin, in which this adhesive capacity is even more pronounced. These events did not change VLA-6 expression on adhered thymocytes. It has been described [12] that thymocytes obtained from GH-Tg and GH-injected BALB/c mice adhere in higher numbers onto laminin than thymocytes from wild-type mice. Nevertheless, membrane levels of laminin receptor VLA-6 on thymocytes did not change. Thus, our results extend actions of GH, with respect to the adherence of human thymocytes to laminin substrate, and demonstrate that the presence of this hormone also stimulates cell adhesion to a generic substrate (BSA).

Regarding the unchanged VLA-6 expression, Vielkind et al. [23] conceptualize that integrins have low activity in resting cells but can be stimulated to mediate adhesion in response to chemokines and cytokines. These stimuli induce a process of “inside-out” signaling that is typically associated with increased avidity of the integrin by its ligand (as the result of clustering of receptors and their greater lateral mobility) and increases its affinity. Our results demonstrate that exposure of thymocytes to GH does not alter expression of VLA-6 integrin on the plasma membrane. However, as referenced above, it is not enough to check the number of receptors on cell surface, but is necessary to analyze activation state and avidity of these molecules. Since cells with high numbers of receptors can have them in the resting state, even a small number of active receptors on the membrane can generate evident cellular responses.

Cell migration is a complex process that involves many variables. For instance, egress of single-positive cells from thymus to periphery requires that cells must be responsive to signals from blood and secondary lymphoid organs. To respond to these stimuli, varied gene expression of chemokine receptors, adhesion molecules, cell signaling molecules and cytoskeletal reorganization is necessary. Therefore, a number of changes are essential for cell migration [24]. GH has been previously implicated in migration of several types of immune cells, including human monocytes [25] and resting or activated human T cells [26]. Furthermore, GH has ability to serve as a chemotactic signal for macrophages and stimulate their migration in vitro [27].

In this study, GH positively modulated human thymocyte migration on laminin substrate, but not on BSA substrate. It raises the notion that the general effect of GH on thymocyte migration results, at least, from a combined action of ECM in addition to chemokines and other soluble factors in thymus. In murine thymocyte transmigration, GH only increases the number of migrating cells when combined with CXCL12 chemokine, but no alteration in thymocyte migration is observed after GH treatment individually, suggesting that this hormone enhances sensitivity of these cells to migratory stimuli, as well on substrata (in this case, laminin) that are needed for cell migration [28]. The fact that cell migration is stimulated further when GH is combined with laminin is consistent with the observations of Yanagawa et al. [29], who demonstrate that chemotactic activity of stromal cell-derived factor-1α (SDF-1α) is considerably and selectively enhanced in the presence of laminin.

Thymic microenvironment is composed mainly by epithelial cells, which form a meshwork to provide mechanical support and stimuli for proliferation and development of thymocytes. Moreover, macrophages, dendritic cells, fibroblasts and extracellular matrix constitute this network [13, 30]. TEC produces soluble thymocyte modulators including thymic hormones and cytokines. Additionally, TEC/thymocyte interactions occur through classical adhesion molecules. TEC can bind and interact with maturing thymocytes by extracellular matrix ligands and respective receptors. Laminin is distributed heterogeneously within thymic parenchyma, being produced by TECs [13]. Our results showed augmented production of laminin by TEC after GH-treatment. This finding corroborates the idea that this network of epithelial cells is controlled by extrinsic circuits such as neuroendocrine axis. For instance, the augmentation of ECM expression as seen, not only in cultures of TEC lines, but also in primary cultures of epithelial cells derived from isolated thymic nurse lymphoepithelial complexes [11], and in mice overexpressing GH (GH-Tg) that exhibit enhanced laminin deposition in cortical and medullary regions of thymic lobules [12].

Considering these effects of GH on human thymocytes and TEC separately, we observed its effects on TEC/thymocyte co-culture. In this assay, we demonstrated two main aspects. First, as shown by immunocytochemistry, GH stimulates laminin production by TEC, and this phenomenon is directly related to thymocyte adhesion and migration, as shown by our initial results. Thus, GH contributes to intrathymic differentiation that occurs concomitantly with thymocyte adhesion and migration through thymic stroma [18]. The second observation is fluctuation in subsets, which is displayed after 24 h of contact with TEC, in the presence of GH.

We hypothesize that GH may act indirectly promoting secretion of interleukin-7 (IL-7) by TEC. GH may act directly upon immune tissues or its effects may be mediated indirectly through insulin-like growth factor-1 (IGF-1) [8, 9]. Reduced serum concentration of IL-7, smaller thymic volume and diminished output of naive T-lymphocytes observed in GH deficiency children suggest that thymic function may be impaired in the presence of defective GH production. In these children, GH deficiency was associated with a pattern of impaired synchronous release of GH and IGF-1 [31]. Furthermore, GH treatment enhances circulating levels of IGF-1 and IL-7 of HIV-1–infected adults. These findings suggest that IGF-1 occupies an important role in GH-mediated enhancement of T cell production and offer fundamental insight into the mechanism of GH effects on the human immune system [32]. More recently, IGF-1, a proximal mediator of the metabolic action of GH, was found to induce expression and release of IL-7 in cultured human thymic epithelial cells [33].

The role of IL-7 in thymopoiesis is multifaceted, promoting thymocyte proliferation and survival by upregulating anti-apoptotic protein Bcl-2 and downregulating pro-apoptotic protein Bax [34]. GH can modulate genes committed to distinct biological activities of thymic epithelial cells, including expression of this cytokine [35].

IL-7 receptor is expressed on DN thymocytes but is downregulated in DP thymocytes and is restored after positive selection, when thymocytes complete differentiation process (SP thymocytes) [36]. DN and both SP subsets thymocytes were responsive to IL-7 and DP population responded poorly [37]. If TEC increases IL-7 secretion because of GH treatment, these thymocyte subsets (DN, CD4^+^SP and CD8^+^SP) would display increased proliferation, which would explain their higher absolute number, whereas the numbers of DP thymocytes decreases in comparison to control group.

Considering all these data, we suggest that GH has pleiotropic effects on lymphoid (thymocytes) and non-lymphoid (TEC) compartments of thymus, thus corroborating with physiology of this organ in supplying T cells to the organism. This study also contributes to current information about effects of GH on human thymic cells, which is important when proposing GH-based therapies for the recovery of the immune system.

## Conclusions

These results point that GH is involved in adhesive and migratory capacity of human thymocytes and modulates production of laminin by TEC, without altering VLA-6 expression on both cells. Furthermore GH positively increases absolute number of double-negative (CD4^−^CD8^−^) thymocytes and single-positive CD4^+^ and CD8^+^ thymocytes after co-culture with TEC in vitro. In this context, considering that GH has been used as an adjuvant therapeutic agent in immunodeficiencies, these data presented herein provide background knowledge for future GH-based immunotherapy interventions.

## Methods

### Reagents and antibodies

Recombinant human GH was purchased from Dong-A Pharmaceutical Co., Ltd (Dalseong-Gun, Daegu, South Korea). Laminin 111 protein, primary anti-laminin antibody and secondary goat anti-rabbit-FITC conjugated antibody were purchased from Sigma-Aldrich (St. Louis, MO, USA). Monoclonal antibodies: anti-CD4 allophycocyanin (APC), anti-CD8 peridinin chlorophyll protein (PerCP), anti-CD49f phycoerythrin (PE) and rat IgG2a PE were purchased from eBioscience (San Diego, CA, USA). Fetal bovine serum (FBS), RPMI-1640 medium, l-glutamine, gentamicin, Trypsin–EDTA, phosphate buffered saline (PBS) and bovine serum albumin (BSA) were obtained from Sigma-Aldrich.

### Cells and culture conditions

Human thymic fragments were obtained from children (aged 1 day to 9 years) undergoing corrective cardiac surgery at Santa Casa de Misericórdia Hospital. Tissue was minced, large aggregates were removed by passing through a nylon mesh and thymocytes in suspension were kept in RPMI-1640 supplemented with 10 % heat-inactivated FBS, 1 % l-glutamine and 40 mg/mL gentamicin, at 4 °C before the experiments. All procedures were approved by the ethics committee on research of UFAL by protocol number 028370/2010-07.

Human thymic epithelial cell line (TEC), generated from explants of a postnatal organ [38], was utilized in this study. Cell cultures were grown in a complete RPMI-1640 medium, at 37 °C in a humidified 5 % CO_2_ atmosphere. Passage of cells by trypsin–EDTA treatment was performed after every 3–4 days.

### Thymocyte adhesion assay

To assess thymocyte adhesive capacity, 6-well culture plates were coated for 1 h at room temperature with 10 µg/mL laminin or BSA as a control. In these assays, 1 × 10^7^ fresh thymocytes were allowed to adhere on substrata for 1 h, in the presence or absence of GH (100 ng/mL) and 3 mL RPMI/FBS 2 %, at 37 °C. After removal of non-adherent thymocytes by PBS washing, adherent thymocytes were harvested with cool PBS, counted and analyzed by flow cytometry.

### Thymocyte migration assay

Thymocyte migratory activity was evaluated using a transwell system (6.5-mm diameter, 5-μm pore size, polycarbonate membrane) (Corning Costar, Cambridge, MA, USA) using cell suspensions. In this chemotaxis assay, membranes were coated on both sides with 10 µg/mL laminin or BSA for 1 h at 37 °C, followed by 1 h of blocking with 1 % BSA. Thymocytes (2.5 × 10^6^) in 100 µL 0.5 % BSA/RPMI 1640 with or without GH (100 ng/mL) were then added to the upper chamber, and 600 µL 0.5 % BSA/RPMI 1640 was added to the lower chamber. After 3 h of incubation at 37 °C in 5 % CO_2_-containing air, cells migrated into the lower chambers and were removed, counted and analyzed by flow cytometry.

### Cytofluorometry

Thymocyte suspensions in PBS with 4 % FBS were incubated with a mixture of appropriate dilutions of the following mAb: anti-CD4/APC, anti-CD8/PerCP, anti-CD49f/PE, as well as, isotype-matched negative controls for each fluorochrome. Cells were stained for 20 min at 4 °C, fixed with PBS/Formaldehyde 2 % and analyzed by flow cytometry on FACSCanto II (BD Biosciences). A gate excluding cell debris and nonviable cells was applied using forward *versus* side scatter parameters pre-established for thymocytes. Analysis with FACSDiva software (BD Biosciences) was performed after recording 10,000–50,000 events for each sample.

TEC were grown in 6-well plate (3 × 10^5^ cells/well) and treated with GH (100 ng/mL) for 24 h, in 500 µL RPMI/FBS 2 %. Cells were recovered by scraping and incubated with diluted antibody (anti-CD49f) or isotype-matched negative control. After washing, the cells were fixed with PBS/Formaldehyde 2 % and analyzed by flow cytometry.

### Immunocytochemistry

Human TEC (5 × 10^3^) were plated in a 8-well Lab-tek chamber slides, with complete medium for 16 h to allow cell adhesion. The medium was then replaced and cells were treated with GH (100 ng/mL). After 24 h, cultures were washed with PBS and fixed with methanol, followed by incubation with anti-laminin primary antibody for 1 h at room temperature, washed with PBS, and incubated with goat anti-rabbit-FITC conjugated secondary antibody for 45 min at room temperature. Immunostained samples were analyzed by fluorescence microscopy (Nikon Eclipse 50i; Nikon Instruments Inc., Chicago, IL, USA). A negative control with secondary antibody was used and did not show any significant immunolabeling. Quantitative fluorescence analysis were performed by transforming specific staining to pixels and by dividing the total pixel numbers by the area analyzed, thus obtaining the number of pixels/μm^2^, using the ImageJ software.

### TEC/thymocyte co-culture assay

Co-culture assays were performed in 24-well plates at a ratio of 100 thymocytes/TEC. Firstly, 1 × 10^4^ TEC were plated in a well with 500 µL complete RPMI and after 16 h of cell adhesion, 1 × 10^6^ fresh thymocytes were plated on the TEC monolayer while replacing the medium with 500 µL RPMI/FBS 2 % with or without GH. After 24 h of co-culture, thymocytes were recovered, counted and analyzed by flow cytometry.

### Statistical analysis

The data obtained were analyzed using one-way ANOVA followed by Newman-Keuls post-test or by Student’s t test and considered as statistically significant when p values were ≤0.05. Values were presented as the mean ± standard error of the mean (SEM).
